# Case Report: Report of 2 cases of periprosthetic joint infection caused by Brucella after joint replacement and literature review

**DOI:** 10.3389/fsurg.2026.1789336

**Published:** 2026-05-22

**Authors:** Yaxing Ma, Jun Li, Tao Ma, He Shang, Xueqi Liu, Tianxiang Yang, Jinpeng Liang, Biao Ma, Ruoyu Wang, Desheng Chen

**Affiliations:** 1 Department of Orthopedics, Third Clinical Medical College of Ningxia Medical University, Yinchuan, China; 2 Department of Joint Surgery, Ningxia Hui Autonomous Region People’s Hospital (Affiliated Hospital of Ningxia Medical University), Yinchuan, China

**Keywords:** Brucella, clinical diagnosis, periprosthetic joint infection (PJI), total knee arthroplasty (TKA), treatment regimens

## Abstract

**Objective:**

To explore the clinical characteristics, diagnostic methods, and therapeutic strategies of Brucella-induced periprosthetic joint infection (PJI) after total knee arthroplasty (TKA), so as to provide references for clinical diagnosis and treatment.

**Methods:**

The clinical data of 2 patients with Brucella-induced PJI after TKA were analyzed retrospectively. One patient had an insidious onset 8 years after TKA, presenting with progressive joint pain and low-grade fever; the other had an acute onset 1 month after TKA, with wound redness, swelling, and exudation as the main symptoms, and initial negative bacterial culture. The key points of diagnosis and treatment were summarized by integrating epidemiological history, laboratory examinations, imaging findings, and the efficacy of surgical intervention.

**Results:**

For patients with a chronic onset and no prosthetic loosening, long-term anti-infective therapy with ceftriaxone sodium combined with doxycycline is administered. For those with an acute onset, debridement and implant retention (DAIR) surgery is performed, followed by anti-infective therapy with ceftriaxone sodium combined with rifampicin. After treatment, the inflammatory markers of the two patients returned to normal, the titer of the Brucella agglutination test decreased to the normal range, and the knee joint range of motion was restored.

**Conclusion:**

Brucella-induced PJI has atypical clinical manifestations and is easily confused with aseptic loosening and synovitis. The acute case received DAIR while the chronic case was treated with antibiotics alone。Diagnosis should emphasize the history of exposure to pastoral areas or consumption of undercooked livestock products. Preliminary experience suggests that extended culture, Brucella PCR, and adjunctive SPECT/CT may help improve diagnostic yield. Surgical intervention combined with long-term sensitive antibiotic therapy and prolonged follow-up for at least 12 months is the preferred treatment strategy. This report can serve as a reference for the diagnosis and treatment of such rare infections in livestock-farming regions.

## 1 Introduction

Total knee arthroplasty (TKA) is the gold standard for the treatment of end-stage knee joint diseases (such as severe osteoarthritis and rheumatoid arthritis). It can significantly relieve joint pain, restore joint function, and improve patients’ quality of life. However, periprosthetic joint infection (PJI), as one of the most serious complications after TKA, has an incidence rate of approximately 1%–2%. Once it occurs, it not only leads to surgical failure but may also cause chronic pain, joint deformity, and even sepsis due to infection spread, increasing the morbidity and mortality of patients and imposing a heavy medical burden. In recent years, with the annual increase in the number of TKA surgeries, the incidence risk of Brucella-induced PJI after TKA has shown an upward trend (1). Nevertheless, the current clinical understanding of this disease remains insufficient, and there is a lack of unified diagnostic and therapeutic standards. Some clinicians neglect epidemiological history and fail to adopt targeted laboratory examinations, resulting in a high rate of missed diagnosis and misdiagnosis (2). Based on this, this study retrospectively analyzed the diagnosis and treatment process of 2 patients with Brucella-induced PJI after TKA. Combined with relevant domestic and foreign literature, the clinical characteristics, key diagnostic points, and therapeutic strategies of this disease were summarized, aiming to improve clinicians’ ability to identify this disease, optimize the diagnosis and treatment process, and improve patient prognosis.

## 2 Case presentation

### 2.1 Case 1

A female patient underwent left total knee arthroplasty (TKA) in our hospital in 2015 due to severe left knee osteoarthritis. Postoperatively, her function recovered well with no obstacles in daily activities. In 2023, she developed left knee joint pain without clear incentives. In 2024, she gradually presented with local swelling, increased skin temperature, limited motion, and intermittent low-grade fever (37.3–38.0℃). Symptoms were not relieved by NSAIDs or physical therapy. Given her 5-year history of residence in a pastoral area, long-term contact with cattle and sheep, and occasional consumption of undercooked lamb, Brucella infection was highly suspected. In August 2024, the patient underwent Brucella agglutination test in another hospital, which was positive (1:400 +++), and the titer was rechecked as 1:1600 + in another hospital. She was diagnosed with “brucellosis” and received antibiotic treatment (specific regimen unknown), after which the symptoms were relieved. In February 2025, the rechecked titer decreased to 1:50 +, but she still complained of left knee discomfort. The symptoms recurred after oral administration of rifampicin and other drugs, so she was admitted to our department for further diagnosis and treatment. She had no history of chronic diseases such as hypertension and diabetes, but had a 5-year history of residence in pastoral areas, with long-term contact with cattle and sheep, and occasional consumption of undercooked lamb.

#### 2.1.1 Physical examination

A localized swelling is visible on the lateral aspect of the left knee joint. Multi-point symmetric skin temperature measurement was performed at 6 cm, 4 cm, 2 cm above the knee, at the joint line, and 2 cm, 4 cm, 6 cm below the knee. The skin temperature at the affected knee joint was 37.6℃, 1.7℃ higher than the contralateral side, indicating focal infectious inflammation (Table 1). This multi-point measurement is a routine standardized assessment for periprosthetic joint infection in our department, helping distinguish infection from mechanical pain. Tenderness is positive around the joint, joint range of motion is limited, the longitudinal compression test is positive, and the patellar tap test is negative.

**Table 1:** Patient skin temperature (℃).

Distance from the knee joint	The affected lower limb	The contralateral lower limb
6 cm above the knee	36.0	36.0
4 cm above the knee	36.2	35.9
2 cm above the knee	36.2	36.0
At the knee joint	37.6	35.9
2 cm below the knee	36.9	35.9
4 cm below the knee	35.9	35.7
6 cm below the knee	35.7	35.7

#### 2.1.2 Imaging examinations

**Figure 1:**
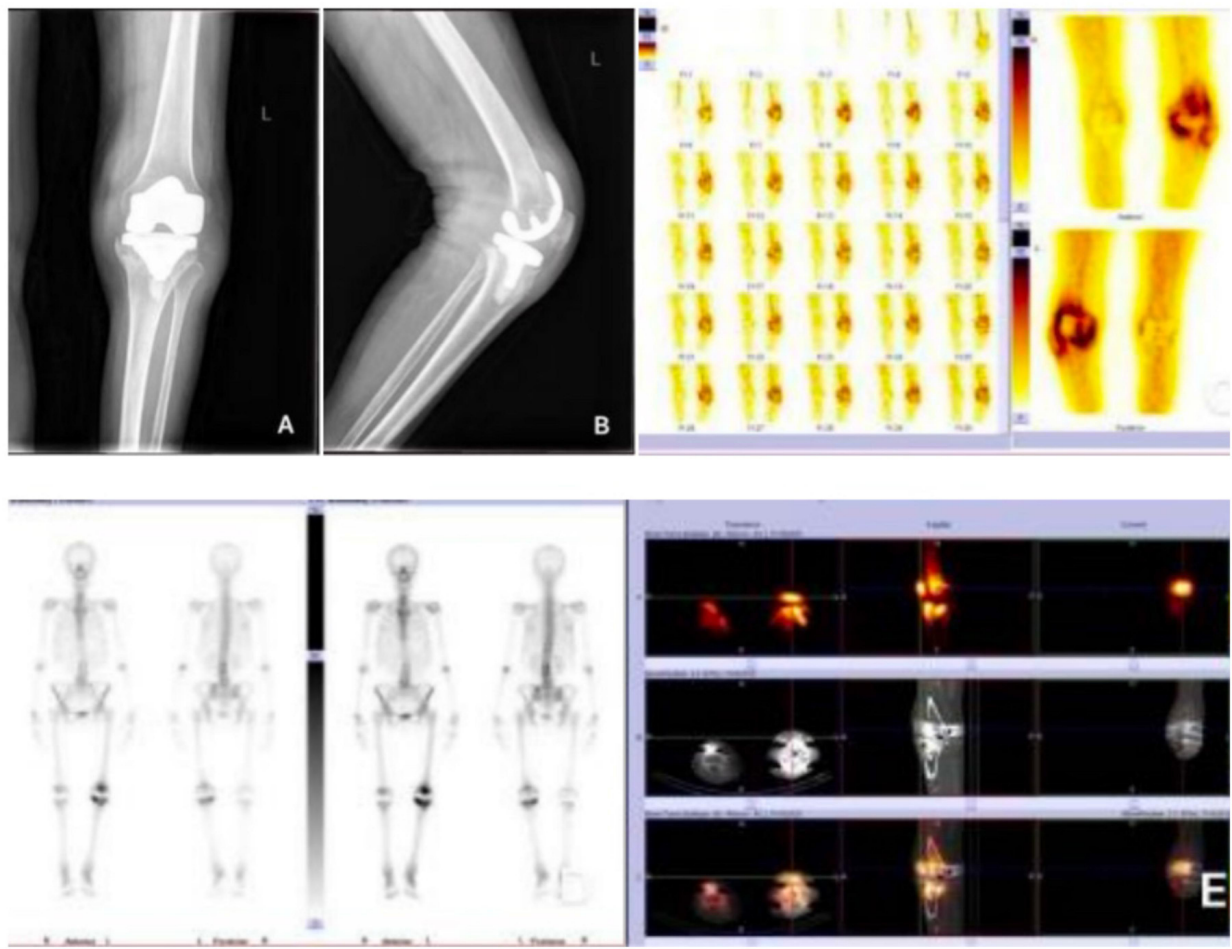
**(A,B)** Anteroposterior and lateral x-ray films of the left knee joint 10 years after total knee arthroplasty; **(C)** dynamic imaging of both lower extremities; **(D)** whole-body bone scan; **(E)** SPECT/CT tomographic fusion imaging.

Post-admission anteroposterior and lateral x-rays of the knee joint (Figures 1A,B) showed periprosthetic lucency and osteolysis, consistent with prosthetic loosening. Dynamic imaging of both lower extremities (Figure 1C): The large arteries of both lower extremities began to opacify 2 seconds later with clear visualization; abnormal radioactive uptake was observed in the left knee joint on the flow phase and blood pool phase; a radioactive defect area was seen in the right knee joint. Whole-body bone scan (Figure 1D): All bones of the body were basically clearly visualized with uneven radionuclide distribution; both knee joints showed post-replacement changes; increased radioactive uptake was observed in the left knee joint. SPECT/CT tomographic fusion imaging (Figure 1E): Metal internal fixation shadows were seen in both knee joints, with good position and shape of the internal fixation and no signs of fracture; the bone density of the distal left femur and proximal left tibia was uneven, accompanied by swelling of the surrounding soft tissues, and increased radioactive uptake was observed in the corresponding areas. No abnormal radioactive uptake was found in other parts.

#### 2.1.3 Laboratory examinations

Erythrocyte sedimentation rate (ESR) was 121.09 mm/h; high-sensitivity C-reactive protein (hs-CRP) was 61.34 mg/L; Brucella agglutination test was 1:400 +++; Rose Bengal plate test (RBPT) was positive; tuberculosis infection T-cell spot test (T-SPOT.TB) was negative; Enterococcus faecium was isolated only from superficial wound swabs. Joint fluid and deep tissue cultures were negative for this organism. It was considered a superficial contaminant and not related to the periprosthetic infection. Brucella PCR was positive in joint fluid.

#### 2.1.4 Preliminary diagnosis

Postoperative state of left knee arthroplasty; Periprosthetic joint infection (Brucellosis-associated infection).

#### 2.1.5 Treatment

**Figure 2:**
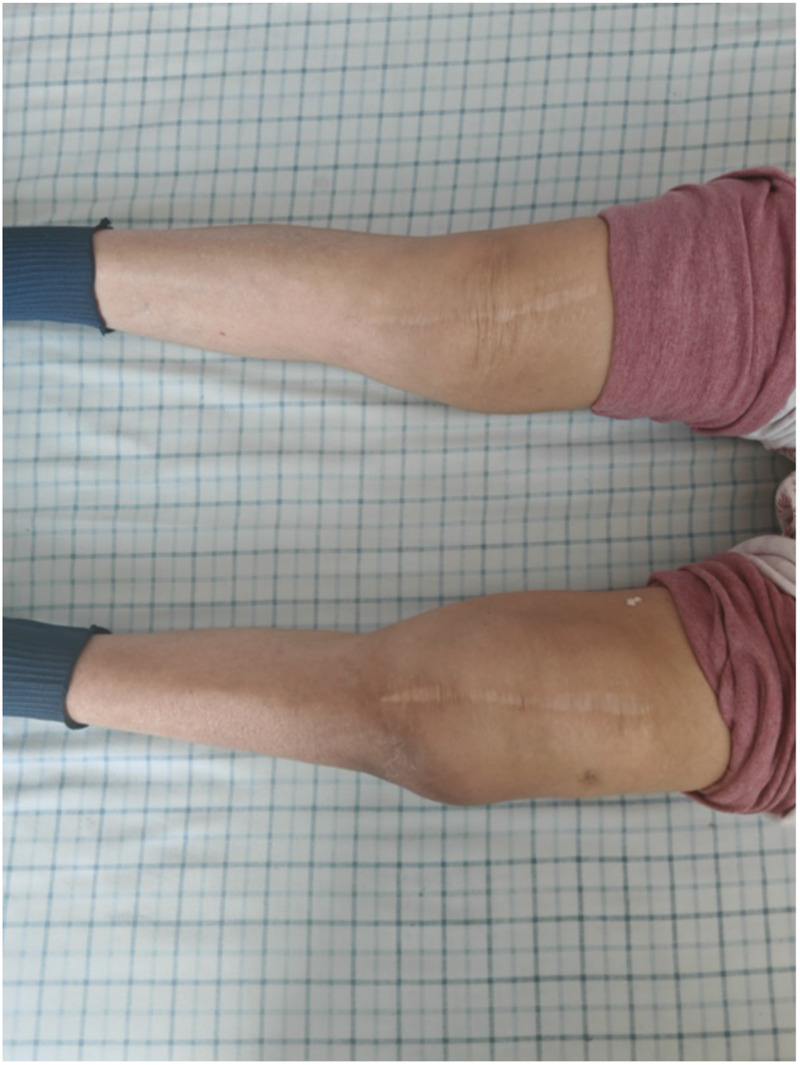
Comparison of the affected lower limb and the contralateral lower limb in the patient two weeks after treatment.

The patient had a chronic indolent infection (> 1 year), no severe osteolysis or soft tissue necrosis, and the prosthesis was relatively stable; therefore, DAIR was not performed. The patient received intensive intravenous therapy with ceftriaxone sodium plus doxycycline for 2 weeks, followed by oral doxycycline plus rifampicin for 12 weeks, with a total course of 14 weeks. After 2 weeks of treatment, the swelling of the affected lower extremity subsided significantly (Figure 2). At the 6-month follow-up, the patient remained free of pain, swelling, or fever. ESR and hs-CRP were normal. Brucella agglutination titer decreased to 1:50 (negative). Knee range of motion recovered to 0–110°. No progression of prosthetic loosening was observed, and daily activities were unrestricted.

### 2.2 Case 2

**Figure 3:**
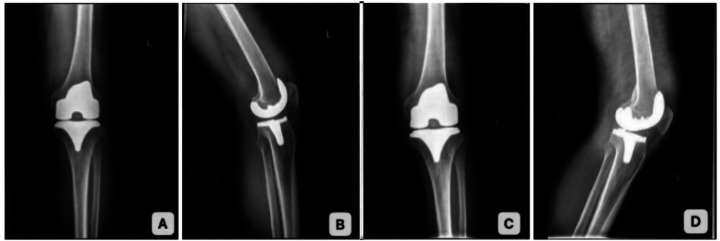
**(A,B)** Anteroposterior and lateral x-ray films of the left knee joint after total knee arthroplasty; **(C,D)** anteroposterior and lateral x-ray films of the left knee joint with infection after total knee arthroplasty.

A male patient underwent left total knee arthroplasty in August 2025. He developed wound redness, swelling, and purulent discharge at 15 days postoperatively. He was admitted to our hospital in September 2025, 1 month after TKA and 10 days after symptom onset, due to persistent left knee pain and wound discharge. The patient and his family reported that he underwent left total knee arthroplasty (TKA) in our hospital 1 month prior, with good postoperative recovery (Figures 3A,B). Ten days before admission, he developed wound redness, swelling, and purulent discharge accompanied by pain, without obvious lower limb numbness, radiating pain, chest tightness, palpitations, cough, expectoration, nausea, vomiting, or other discomfort. He was initially seen in our department at that time. The wound discharge was superficial without deep fluctuation, and the patient refused joint aspiration. Oral cephalosporin antibiotics were administered for 5 days. Conservative treatment was chosen because no sinus tract was found, exudate was limited, and inflammatory markers were only mildly elevated. For further diagnosis and treatment, he visited the outpatient clinic of our hospital again. After detailed inquiry of medical history, physical examination, and relevant auxiliary examinations. At the outpatient visit, a sinus tract communicating with the joint cavity, purulent drainage, and local inflammatory signs were confirmed. The patient was immediately admitted for emergency surgery. He had a 10-year history of hypertension, which was controlled with oral candesartan cilexetil tablets, and his blood pressure was maintained at approximately 130/80 mmHg. He had a history of consuming undercooked beef 1 month before the onset of symptoms.

#### 2.2.1 Physical examination

Physiological curvature of the spine was preserved; the left knee joint showed flexion deformity and moderate swelling; no varicose veins were found in both lower extremities; the skin of the left knee was red and swollen, with ulceration and sinus tract formation, and purulent discharge was observed; the local skin temperature of the left knee joint was significantly increased; tenderness around the left knee joint was positive.

#### 2.2.2 Hematological examinations

Erythrocyte sedimentation rate (ESR) was 48.5 mm/h, and high-sensitivity C-reactive protein (hs-CRP) was 21.22 mg/L. No bacterial growth was detected in necrotic tissue culture and joint aspiration culture. The postoperative tissue culture result was Brucella spp. Three days after surgery, ESR and hs-CRP returned to the normal range.

#### 2.2.3 Imaging examinations

Anteroposterior and lateral x-rays of the knee joint (Figures 3C,D) showed the left knee prosthesis was in good position without loosening, displacement, or osteolysis; the periprosthetic soft tissue density was mildly increased, with obvious swelling and thickening; no periosteal reaction, bone destruction, or abnormal calcification was found; the joint space was preserved, and the bone structure around the prosthesis was intact.

#### 2.2.4 Treatment

The patient presented with acute infection (< 3 weeks postoperatively), a well-fixed and stable prosthesis without loosening or osteolysis, and a localized sinus tract that could be completely excised, which met the revised criteria of the 2025 ICM and EBJIS. Intraoperative findings included: a wound at the distal end of the original surgical incision with bloody exudation, a sinus tract extending deep to the bone and communicating with the joint cavity; local soft tissues appeared pale, degenerated, necrotic, and poorly perfused; obvious bloody fluid exuded from the joint cavity when the knee joint was moved. The sinus tract was completely excised down to the bone surface. The skin, subcutaneous tissue, and deep fascia were incised layer by layer along the original incision; the joint capsule was incised along the medial edge of the patella, and the patella was everted to expose the joint cavity. The continuity and function of the medial and lateral collateral ligaments were intact. The tibial insert was removed, thorough debridement was performed, and tissues with poor blood supply were resected. After satisfactory debridement, the joint cavity was soaked in povidone-iodine and thoroughly irrigated. After re-sterilization, the polyethylene tibial insert was exchanged (size E, thickness 8 mm). The joint was reduced, and the prosthesis was confirmed to be well-fitted, with stable joint and appropriate tension. Repeated irrigation was performed, 2 drainage tubes were indwelled, 1 g of vancomycin was locally applied in the joint cavity, which is a routine practice for acute periprosthetic joint infection in our department to achieve high local antibiotic concentration and inhibit biofilm formation, and the incision was sutured layer by layer. Tranexamic acid was injected into the joint cavity.

## 3 Discussion

### 3.1 Clinical characteristics and diagnostic difficulties of Brucella-induced periprosthetic joint infection

Periprosthetic joint infection (PJI) after total knee arthroplasty (TKA) is one of the most severe complications, with an incidence rate of approximately 1%–2%. Common pathogenic bacteria include Gram-positive bacteria such as Staphylococcus aureus and coagulase-negative staphylococci. However, PJI caused by Brucella is extremely rare, with fewer than 50 cases reported worldwide. Its clinical characteristics differ significantly from those of conventional bacterial PJI, posing substantial challenges to diagnosis (3).

Based on the two cases in this study, Brucella-induced PJI can present with two distinct onset patterns: Case 1 was an insidious onset type, with symptoms appearing 8 years after TKA. Initial manifestations included non-specific joint pain and low-grade fever, with atypical local inflammatory signs. The patient sought medical attention multiple times without a clear diagnosis until a positive Brucella agglutination test raised suspicion of infection. Case 2 was an acute postoperative infection type, with wound redness, swelling, and exudation occurring 1 month after TKA. The onset was acute, but initial bacterial culture was negative, making it easily confused with postoperative aseptic inflammation or other infections (4). Common characteristics of both onset patterns are as follows:1. Inflammatory indicators: Erythrocyte sedimentation rate (ESR) and high-sensitivity C-reactive protein (hs-CRP) were significantly elevated (Case 1: ESR 121.09 mm/h, hs-CRP 61.34 mg/L on admission; Case 2: ESR 48.5 mm/h, hs-CRP 21.22 mg/L on admission). However, the total white blood cell (WBC) count and neutrophil ratio in routine blood tests were mostly within the normal range or slightly elevated. This stands in stark contrast to conventional bacterial PJI, which is typically accompanied by a WBC count > 10 × 10⁹/L and a neutrophil ratio > 75%. The core reason is that Brucella is an intracellular pathogen that primarily invades macrophages and synoviocytes, replicates intracellularly, and induces a chronic inflammatory response. It has a weak stimulatory effect on peripheral blood leukocytes, thus not causing a significant increase in WBC count (5). 2. Imaging findings: Early knee x-rays mostly only show soft tissue swelling without signs of prosthetic loosening or osteolysis (e.g., x-rays of Case 2 1 month after surgery showed a well-positioned prosthesis with only slightly increased radiodensity of surrounding soft tissues). In the later stage, as bacteria erode bone and the prosthesis, periprosthetic circumferential lucency and osteolysis may occur (e.g., x-rays of Case 1 on admission showed circumferential lucency on the tibial and femoral sides and medial tibial plateau osteolysis). However, these findings are highly overlapping with aseptic loosening after TKA, and infection cannot be distinguished from non-infectious factors based solely on x-rays. SPECT/CT is not a first-line diagnostic tool for PJI per current guidelines, but it was used as an adjunctive modality in our cases: it can not only clarify the prosthesis position and osteolysis range but also assess inflammatory activity through increased radioactive uptake (SPECT/CT of Case 1 showed increased radioactive uptake in the distal femur and proximal tibia, confirming infectious inflammation; in contrast, aseptic loosening usually has no obvious radioactive uptake), which is a key method to distinguish the two (6). 3. Pathology: The positive rate of bacterial culture is low, and routine bacterial culture (5–7 days) is mostly negative (initial cultures of both cases showed no bacterial growth). Brucella requires biosafety level 3 (BSL-3) handling, a microaerophilic environment with 5%–10% CO₂, an optimal temperature of 35–37°C, special selective media, and extended culture for 14–21 days. Brucella-specific PCR was positive before culture conversion in both cases, enabling early diagnosis. If the laboratory does not follow the dedicated culture protocol, false-negative results are likely to occur, which is also an important reason for delayed diagnosis of Brucella-induced PJI (7).

#### 3.1.1 Diagnostic difficulties mainly manifest in three aspects

First, epidemiological history is easily overlooked. Brucellosis is mostly associated with contact with livestock such as cattle and sheep or consumption of undercooked livestock products, including direct contact with excreta of infected livestock and consumption of undercooked beef, mutton, and dairy products. If the patient has no clear history of residence in pastoral areas or direct contact, clinicians are prone to missing this diagnosis (8). Second, clinical manifestations are atypical. Joint pain and limited mobility in patients with insidious onset are easily confused with aseptic loosening and synovitis, without specific systemic symptoms. Wound redness, swelling, and exudation in patients with acute onset are easily confused with poor postoperative wound healing or other bacterial infections (9). Third, laboratory diagnostic methods are limited. Routine bacterial culture is difficult to detect Brucella, and the titer of Brucella agglutination test fluctuates significantly. Dynamic observation combined with clinical symptoms is required to avoid excluding infection based solely on a single low-titer result (10).

According to the 2018 International Consensus Meeting on Musculoskeletal Infection (ICM, Philadelphia) and European Bone and Joint Infection Society (EBJIS) 2025 updated criteria, the diagnosis of PJI is based on unified major and minor criteria, which have replaced the earlier 2013 MSIS/ICM guidelines. However, for Brucella, the culture protocol needs to be adjusted to improve the detection rate (11). For patients suspected of Brucella-induced PJI, in addition to routine bacterial culture, the culture time should be extended to 14 days, and special Brucella culture media should be used. Since antibiotics can inhibit bacterial activity and lead to negative culture, if the patient has already used antibiotics, specimens should be collected 3–7 days after discontinuing antibiotics (12). In addition, Brucella nucleic acid detection is rapid and sensitive, and can be used as a supplementary diagnostic method for suspected cases, especially in patients who have received antibiotic treatment (13). Joint aspiration is the first-line diagnostic procedure for suspected PJI.SPECT-CT and MRI may be used as adjunctive imaging tests when the diagnosis remains unclear. In Case 1 of this study, periprosthetic inflammation was confirmed by SPECT/CT, and the diagnosis was made based on positive Brucella agglutination test and nucleic acid detection. In Case 2, Brucella was detected by extending the culture time, both in line with the above diagnostic ideas. This suggests that clinicians should consider the possibility of Brucella infection when facing unexplained joint pain and elevated inflammatory indicators after TKA, and promptly complete relevant examinations. Both cases met the 2018 MSIS criteria for PJI. Case 1: elevated ESR/CRP, positive Brucella serology and PCR, and imaging evidence of infection. Case 2: sinus tract communicating with the joint, positive pathogen identification after extended culture, and elevated inflammatory markers.

### 3.2 Selection of treatment strategies for Brucella-induced periprosthetic joint infection

WHO and IDSA guidelines recommend doxycycline plus rifampicin as first-line therapy for brucellosis. In our study: Chronic case (no DAIR): Ceftriaxone + doxycycline was used for intensive intravenous treatment of deep osteoarticular infection. Acute case (after DAIR): Ceftriaxone + rifampicin was used for better bone and biofilmpenetration. Both regimens are clinically supported in severe osteoarticular brucellosis. The treatment of Brucella-induced PJI should follow the principle of combining anti-infection therapy with surgical intervention. As an intracellular pathogen, Brucella can invade macrophages and synoviocytes for replication, and conventional antibiotics are difficult to penetrate the cell membrane to exert their effects (14). Moreover, as a foreign body, the prosthesis activates the body's immune response, prompting bacteria to form a protective biofilm on its surface composed of bacteria, extracellular polysaccharides, and proteins, which further hinders antibiotic penetration and leads to refractory infection (15). Therefore, the treatment plan should be comprehensively formulated based on the severity of the patient's infection, prosthesis stability, soft tissue conditions, and systemic status. Currently, the commonly used clinical treatment strategies mainly include two types: “Debridement, Antibiotics, and Implant Retention (DAIR) + long-term anti-infection therapy” and “prosthetic revision + long-term anti-infection therapy”. The indications should be strictly mastered to avoid blind selection of surgical methods leading to treatment failure (16).

For patients with acute onset, short duration of infection, stable prosthesis, no obvious osteolysis or soft tissue necrosis, thorough debridement + prosthetic insert replacement + long-term anti-infection therapy can be attempted (17). Case 2 in this study met the above indications: the infection occurred 1 month after surgery, with a duration of approximately 2 weeks, the prosthesis was well-positioned without loosening, only local soft tissue necrosis was found during surgery, and there was no obvious osteolysis. Therefore, the DAIR procedure was selected, combined with sensitive antibiotic treatment after surgery, achieving good results. It should be noted that the key to the success of the DAIR procedure lies in early debridement and thorough removal of infected tissue. During surgery, the joint cavity should be repeatedly irrigated with a pulse lavage system, and local antibiotics should be placed. After surgery, sensitive antibiotics should be selected based on drug sensitivity test, and the course of treatment is usually 6-12 weeks to avoid infection recurrence due to insufficient course of treatment (18).

For patients with an indolent onset, prolonged infection duration, absence of prosthetic loosening, and presence of significant osteolysis or soft tissue infection, combination therapy with multiple antibiotics for a long course of anti-infective treatment is usually adopted. In contrast, for those complicated with prosthetic loosening, the debridement, antibiotics and implant retention (DAIR) procedure yields a relatively low success rate, and prosthetic revision surgery is generally recommended. This surgery involves thorough debridement, removal of the original prosthesis, implantation of a new prosthesis (one-stage or two-stage revision), and long-term anti-infective treatment (19). In Case 1 of this paper, the infection duration exceeded 1 year without obvious prosthetic loosening. Therefore, long-term anti-infective therapy with ceftriaxone sodium combined with doxycycline was selected. The advantage of one-stage revision is that it can solve prosthetic loosening and infection at one time, reducing the number of surgeries for patients. However, it needs to meet the conditions of thorough debridement, no severe soft tissue defect, and pathogen sensitivity to multiple antibiotics (20). If the patient has severe infection, poor soft tissue conditions, or drug-resistant pathogens, two-stage revision can be considered: the first surgery involves removing the prosthesis and placing an antibiotic-loaded bone cement spacer, followed by anti-infection therapy for 6–8 weeks, and then the second-stage prosthesis implantation to improve the success rate of infection control (21).

The selection of anti-infection drugs should be based on the results of drug sensitivity test. Brucella is sensitive to drugs such as doxycycline, rifampicin, ceftriaxone sodium, and trimethoprim-sulfamethoxazole. Combined drug regimens are commonly used clinically, such as “doxycycline + rifampicin” and “ceftriaxone sodium + doxycycline”, to avoid drug resistance caused by single-drug use (22). For pregnant women, or patients allergic to tetracycline drugs, the regimen can be adjusted to “trimethoprim-sulfamethoxazole + rifampicin”, but close monitoring of adverse drug reactions such as liver injury and hematological abnormalities is required (23). In addition, patients with Brucella-induced PJI need long-term follow-up long-term follow-up for at least 12 months after surgery, with regular re-examinations of inflammatory indicators, Brucella agglutination test, and imaging examinations to alert for infection recurrence. The follow-up time should be at least 1 year. If elevated inflammatory indicators, joint pain, or swelling occur, timely medical attention should be sought to rule out infection (24).

### 3.3 Clinical insights and preventive suggestions

Although Brucella-induced PJI is rare, with the increase in TKA surgery volume and the expansion of Brucellosis endemic areas, its incidence rate is gradually increasing. Clinicians need to improve their understanding of this disease to avoid missed diagnosis or misdiagnosis. Based on the two cases in this study and literature reports, the following clinical insights can be drawn: First, attach importance to the collection of epidemiological history. For patients with unexplained joint pain and low-grade fever after TKA, regardless of whether they have a clear history of contact with pastoral areas, clinicians should inquire about their consumption of undercooked beef, mutton, and dairy products, or contact history with cattle and sheep. If there is relevant exposure history, timely completion of Brucella-related examinations is required to avoid delayed diagnosis due to neglect of epidemiological history (25). Second, optimize the diagnostic process. For patients suspected of PJI, if routine bacterial culture is negative, the possibility of infection with fastidious bacteria such as Brucella should be considered, and the culture protocol should be adjusted in a timely manner, or molecular biological methods such as nucleic acid detection should be used to improve the detection rate. At the same time, combined with functional imaging examinations such as SPECT/CT and MRI to assess periprosthetic inflammatory activity, distinguish aseptic loosening from infectious loosening, and avoid misjudgment (26). The basic timeline and key management information of the two enrolled patients are summarized in Table 2. Third, formulate individualized treatment plans. Based on the patient's infection onset pattern, prosthesis stability, soft tissue conditions, and pathogen drug sensitivity test results, select appropriate surgical methods and anti-infection drug regimens, strictly control the course of antibiotics, and avoid infection recurrence or drug resistance caused by inappropriate treatment plans. Strengthen rehabilitation exercises after surgery to promote the recovery of knee joint function, and closely monitor adverse drug reactions to ensure treatment safety (27).

**Table 2:** Timeline summary.

Key Event	Case 1	Case 2
TKA	2015	August 2025
Symptom onset	8 years post-TKA	1 month post-TKA
Diagnosis	February 2025	September 2025
Treatment	Ceftriaxone + doxycycline (no DAIR)	DAIR + ceftriaxone + rifampicin
Follow-up	18 months	15 months
Outcome	No recurrence	15 months

In terms of prevention, for patients after TKA, especially those living in Brucellosis endemic areas, health education should be strengthened to guide them to avoid consuming undercooked beef, mutton, and dairy products, and avoid contact with infected livestock or their excreta, so as to reduce the risk of Brucella infection. During surgery, strict adherence to aseptic operation principles is required to reduce the risk of surgery-related infections. After surgery, if wound redness, exudation, or joint pain occurs, timely medical attention should be sought to avoid self-medication leading to delayed treatment (28). In addition, for hospitals in Brucellosis endemic areas, Brucella-related examinations can be considered as routine screening items for unexplained PJI after TKA to improve diagnostic efficiency (29).

## 4 Conclusion

In summary, Brucella-induced PJI has atypical clinical manifestations and is difficult to diagnose (30). Given the small sample size, preliminary experience suggests that attention to epidemiological history, optimized diagnostic procedures, and individualized treatment may help improve patient prognosis. Clinicians in brucellosis-endemic areas should maintain a high index of suspicion for this rare condition.

## Data Availability

The datasets presented in this study can be found in online repositories. The names of the repository/repositories and accession number(s) can be found in the article/Supplementary Material.
